# MiR-27a as a diagnostic biomarker and potential therapeutic target in systemic sclerosis

**DOI:** 10.1038/s41598-022-23723-7

**Published:** 2022-11-07

**Authors:** Paria Bayati, Mahsa Kalantari, Mohammad-Ali Assarehzadegan, Hadi Poormoghim, Nazanin Mojtabavi

**Affiliations:** 1 Immunology Research Center, Institute of Immunology and Infectious Diseases, Iran University of Medical Sciences, Tehran, Iran; 2 Department of Immunology, School of Medicine, Iran University of Medical Sciences, Tehran, Iran; 3 Scleroderma Study Group Firuzgar Hospital, Iran University of Medical Sciences, Tehran, Iran

**Keywords:** Rheumatic diseases, Skin diseases, Medical research, Rheumatology

## Abstract

Systemic sclerosis (SSc) or scleroderma is a multiorgan rheumatoid disease characterized by skin tightening or organ dysfunction due to fibrosis, vascular damage, and autoimmunity. No specific cause has been discovered for this illness, and hence no effective treatment exists for it. On the other hand, due to the lack of diagnostic biomarkers capable of effectively and specifically differentiating the patients, early diagnosis has not been possible. Due to their potent regulatory roles in molecular pathways, microRNAs are among the novel candidates for the diagnosis and treatment of diseases like SSc. MiR-27a is a microRNA known for its role in the pathogenesis of fibrosis and cancer, both of which employ similar signaling pathways; hence we hypothesized that Mir-27a could be dysregulated in the blood of individuals affected by SSc and it might be useful in the diagnosis or treatment of this disease. Blood was collected from 60 SSc patients (30 limited and 30 diffuse) diagnosed by a rheumatologist according to ACR/AULAR criteria; following RNA isolation and cDNA synthesis; real-time qPCR was performed on the samples using Taq-Man probes and data were analyzed by the ΔΔCT method. Also, potential targets of miR-27a were evaluated using bioinformatics. It was revealed that miR-27a was significantly down-regulated in SSc patients in comparison to healthy individuals, but there was no difference in miR-27 expression between limited and diffused SSc patients. Besides, miR-27a was found to target several contributing factors to SSc. It seems that miR-27a has a protective role in SSc, and its downregulation could result in the disease's onset. Based on bioinformatics analyses, it is speculated that miR-27a likely targets factors contributing to the pathogenesis of SSc, which are elevated upon the downregulation of miR-27a; hence, miR-27a mimics could be considered as potential therapeutic agents for the treatment of SSc in future studies. Since no difference was observed between limited and diffuse patient groups, it is unlikely that this microRNA has a role in disease progression. According to ROC analysis of qPCR data, miR-27a could be employed as a valuable diagnostic biomarker for SSc.

## Introduction

Systemic sclerosis (SSc) is an inflammatory disease of connective tissue with the hallmark characteristic of over-accumulation of the extracellular matrix, leading to fibrosis in the skin and different organs like lungs and kidneys; as a result, the affected individuals may experience vasculopathy and subsequently organ failure. The patients are placed into two distinct subcategories of limited and diffused, based on their symptoms and skin fibrosis pattern; with the limited form being the less severe, affecting only distal parts of the body and having less internal organs involvement, and the diffused form which affects the trunk and internal organs too^1^. Patients affected with SSc experience a range of symptoms and complications; they are also prone to develop other autoimmune disorders like lupus and cancer^2^. So far, there is no specific cure for this disease, mostly because there are many unknown aspects of this disease, and treatment approaches are symptom-oriented^3^. Because of the wide range of symptoms that SSc bears, diagnostic approaches for this disease are also challenging and the most accepted criteria stated by the ACR/EULAR are also subject to change from time to time. Accordingly, there are different estimations concerning the prevalence and incidence of SSc; for instance, the prevalence of SSc in North America is reported as wide as 13.5 to 44.3 per 100,000 individuals^4–6^. Since conventional treatment approaches are not helpful to cease SSc progression and efficiently ameliorate the symptoms, physicians and scientists consider exploring new approaches such as employing microRNAs in the treatment of SSc^7^. Besides, finding an effective diagnostic biomarker that could facilitate diagnosing SSc at early onset, has been a major interest for researchers^8^. Such a biomarker should be capable of identifying most of the patients or in other words, shows a high profile of sensitivity while also demonstrating a high profile of specificity as well. But currently, the majority of accepted diagnosing criteria for SSc are based on the disease complications rather than laboratory biomarkers; this results in the failure of the very early detection of SSc^9,10^. On the other hand, the available laboratory biomarkers such as auto-antibodies against topoisomeraseI, centromere or RNA polymeraseIII are not found in all SSc patients; besides, they are not much specific and are found in other rheumatoid diseases too. Furthermore, some newer biomarkers such as Lungen-6 (KL-6), surfactant protein-D (SP-D), and CCL18 are limited to the detection of complications such as interstitial lung disease (ILD)^11^. As a result, exploring new approaches such as utilizing microRNAs which could detect the disease rather than the associated complications would be very promising for addressing the issues discussed so far^12^.

MicroRNAs (miRNAs) are non-coding short sequences of ribonucleotides (about 22 nucleotides) that are capable of binding to other RNAs through their 3' UTR, which results in the blockade of protein synthesis from their targets or degradation of those targets^13^. One of the most crucial features of miRNAs is their secretion from the host cell where they were transcribed and processed in the first place; hence they are present not only in the cells but unlike their mRNA targets, they can be found nearly in all of the body fluids including blood and even urine in a highly stable form since they are protected from RNases due to their interactions with proteins^14^. This class of small RNAs is implicated in regulating different aspects of cellular physiology, including proliferation, apoptosis, and different signaling pathways, making them responsible for various abnormalities in cellular functions leading to diseases such as malignancies^15–18^. MiR-27, as its name implies is a microRNA, which is known for contributing to the pathogenesis of cancer, and also it is well known for being involved in adipogenesis^19,20^. One of the key players in fibrosis and associated disorders is TGF-β, which has a central role in the pathogenesis of SSc and almost affects all the aspects of fibrogenesis in different stages as well as different signaling pathways. Accordingly, a previous study has elucidated the role of miR-27 in a mouse model of pulmonary fibrosis as a negative regulator of TGF-βR1 and samd2, which are crucial components of SSc pathogenesis too^21^. It was also demonstrated that TGB-β1 and smad3 are targeted by mir-27 in cervical cancer^22^. Moreover, a different study on lung cancer has reported the concurrent upregulation of miR-27a and downregulation of two significant mediators of the TGF-β signaling pathway, SMAD2, and SMAD4^23^. Among the potent regulators of the TGF-β signaling pathway are PPAR-γ and PTEN, both of which are reported to be targeted by miR-27a^24,25^. Besides, there are some studies regarding the effect of miR-27 on NF-κB which also acts downstream of the TGF-β pathway^26,27^. The hallmark cytokine in fibrosis, interleukin-6 and matrix-metalloproteinase 9 and 13, are also reported to be regulated by miR-27^27^. Moreover, it has been shown that miR-27a expression is also reduced in the lungs of IPF patients and bleomycin-induced lung fibrosis in mice^28^.

There are common molecular pathways in cancer and fibrosis which is a hallmark of SSc, and since there is impaired adipogenesis in patients with SSc and mice models of this disease^2,29–32^; we aimed to evaluate the expression of miR-27a in the whole blood of SSc patients in order to find any deregulations and in case of obtaining a significant result, to test if it is possible to take advantage of miR-27 differential expression as a diagnostic tool.

## Materials and methods

Sixty female SSc patients (30 limited SSc and 30 diffused SSc) presenting to Firouzgar hospital, Tehran, Iran, were enrolled in this study. Male patients were excluded due to their low numbers. The diagnosis of the patients as well as categorizing them into limited and diffused subgroups was done based on ACR/EULAR criteria by an expert rheumatologist. All patients filled out an informed consent form which was confirmed by the ethics committee of the Iran University of medical sciences (all methods were performed in accordance with the relevant guidelines and regulations by the ethics committee of the Iran University of medical sciences; ethic code: IR.IUMS.FMD.REC1396.30675). 20 consented healthy individuals matching the sex and age of the patients' group were also included as controls. Table 1 gives a concise description of the demographic features of the patients. The majority of patients were on a regimen of prednisolone, azathioprine, chloroquine, phosphoesterase inhibitors, along with NSAIDs upon the time blood was drawn.

**Table 1:** Demographic data and representative characteristics of the patients.

Number	Mean age	Sex	Telangiectasia	Renal crisis	PAH^1^	ILD^2^	ARA^3^	TOPO-I^4^	ACA^5^
60	50.95	Female	67.60%	0%	12.67%	44.28%	2.86%	62.86%	1.43%

^1^Pulmonary hypertension, ^2^Interstitial lung disease, ^3^Anti-RNA polymerase III, ^4^Anti-topoisomerase I, ^5^Anti-centromere.

Whole blood was collected from patients each day between 9 am to 12 pm and was immediately added to RNAZOL BD (MRC) (0.5 ml blood with 1 ml RNAZOL), and total RNA was isolated according to the manufacturer's protocol. After evaluating the RNA concentration and purity by NANODROP (Thermofisher); the RNA was converted into cDNA by TaqMan advanced microRNA synthesis kit (Ambion) according to the kit instructions. qPCR was carried out using TaqMan fast advanced microRNA fast master mix (Ambion) and TaqMan advanced microRNA assay (hsa-miR-27a-3p); thermal cycling was performed by RotorGene 6000(Qiagen) as recommended by the manufacturer protocol. hsa-miR-191a assay was employed for data normalization. Data were analyzed using the ΔΔCT method and were compared using ANOVA by GraphPad PRISM software. P-values lower than 0.05 were considered statistically significant.

### Bioinformatics analysis

Using TargetScan, mirpath, mirpathDB, and KEGG pathway, we searched for putative targets of miR-27a which are known to be involved in fibrosis and hence SSc pathogenesis, specifically those contributing to the epithelial to the mesenchymal pathway (EMT).

### Ethics approval and consent to participate

All patients filled out an informed consent form which was confirmed by the ethics committee of the Iran University of medical sciences (ethics code: IR.IUMS.FMD.REC1396.30675). All methods were performed in accordance with the relevant guidelines and regulations by the ethics committee of the Iran University of medical sciences; ethics code: IR.IUMS.FMD.REC1396.30675.

## Results

**Figure 1:**
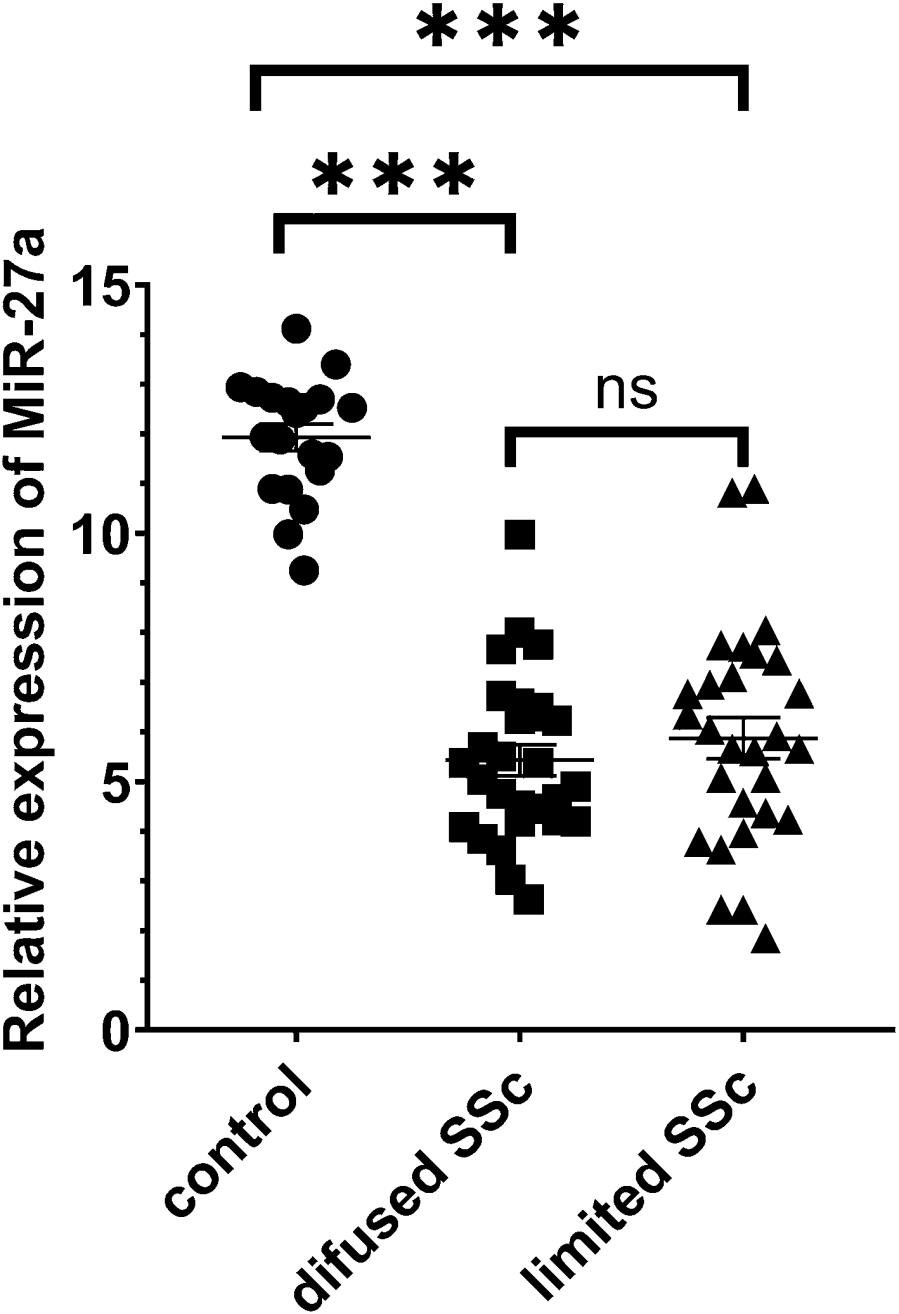
Relative expression of miR-27 in healthy control and patient with diffused or limited systemic sclerosis. Data are shown as mean ± SEM. The expression of miR-27a is downregulated in SSc patients compared with the controls. P-value = 0.0001. There's no significant difference in the expression of miR-27a between diffused and limited SSc patients.

Realtime qPCR data are indicative of miR-27 presence in the whole blood. Comparison of RealTime qPCR data based on the expression of miR-27 between limited and diffused SSc patients and the healthy controls revealed a significant downregulation of miR-27 in both SSc groups compared with the healthy individuals; However, no difference was observed between the limited and diffused SSc patients (Fig. 1).

**Figure 2:**
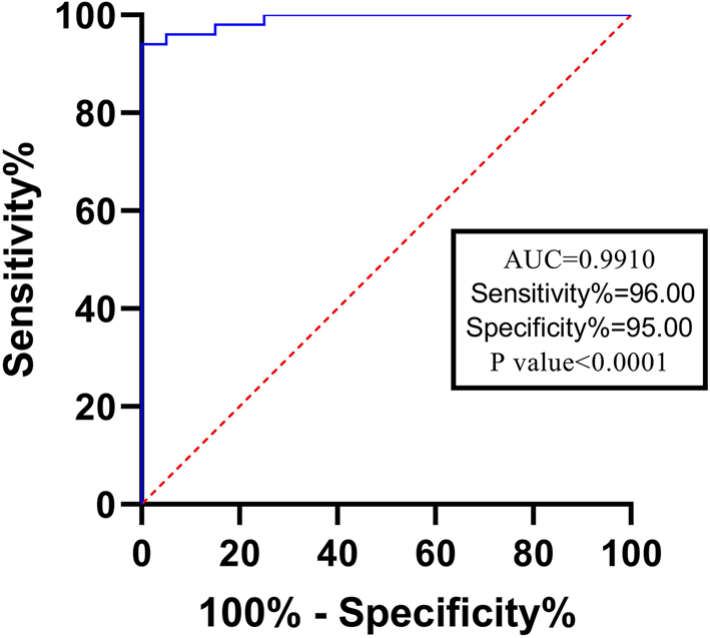
Receiver operative characteristic (ROC) curve of relative expression data for miR-27a with a 0.99 area under the curve illustrates a good differential marker for diagnosing systemic sclerosis patients. P-value = 0.0001, 95% confidence interval = 0.976 to 1.000.

In order to evaluate the predictive value of miR-27 as a diagnostic marker for systemic sclerosis patients, receiver operative characteristic (ROC) analysis was performed and the resulting ROC curve showed a cutoff of < 9.975 (log2 of relative expression) and a good area under the curve (0.99) which is demonstrative of a good differentiating marker. The corresponding sensitivity and specificity of the selected cutoff were 0.96 and 0.95 respectively (Fig. 2).

**Figure 3:**
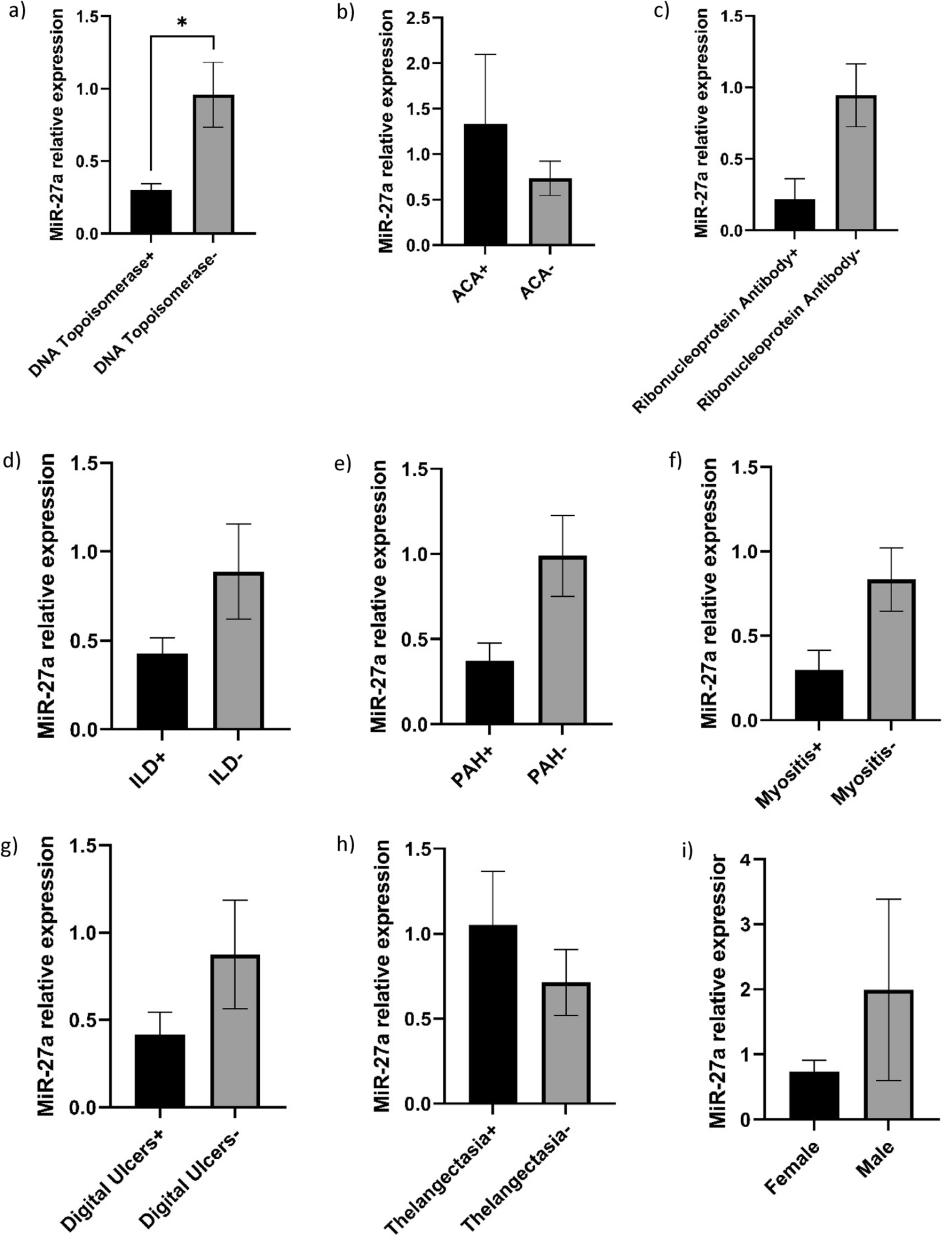
Comparing the expression of miR-27a between different binary situations. (**a**) comparison of miR-27a expression patients positive or negative for DNA topoisomerase (SCL70), (**b**) Comparison of miR-27a expression patients positive or negative for anti-centromere antibody, (**c**) comparison of miR-27a expression patients positive or negative for ribonucleoprotein autoantibody, (**d**) comparison of miR-27a expression patients positive or negative for interstitial lung disease, (**e**) comparison of miR-27a expression patients positive or negative for pulmonary arterial hypertension, (**f**) comparison of miR-27a expression patients positive or negative for myositis, (**g**) comparison of miR-27a expression patients positive or negative for digital ulcers, (**h**) comparison of miR-27a expression patients positive or negative for telangiectasia, and, comparison of miR-27a expression between female and the few male cases.

For better understanding the possible role of miR-27a in the SSc pathogenesis and progression we further conducted a series of analyses in which the expression of miR-27a was compared between groups positive for a specific symptom or a laboratory factor associated with diagnosis or categorization of the disease; it was shown that the downregulation of miR-27a corresponds with the positive SCL70 antibody, while it was associated with negative ACA. Also, the presence of Th/To ribonucleoprotein autoantibody, ILD, PAH, myositis, and digital ulcers are associated with downregulation of miR-27a, but it is associated with lower frequency of telangiectasia. Although the male cases were omitted from the statistical analyses due to their low abundance; here we compared the expression of miR-27a between the female cases and the 5 male cases and it was observed that miR-27a expression is higher in males (Fig. 3).

We also conducted statistical analysis to find correlations between miR-27a expression and patient symptoms like FVC, DLCO, mRSS, CRP, RF and …; although we couldn’t find any significant correlation between miR-27a expression and these symptoms, except for TPO which was found to be significantly corelated with miR-27a (Table 2).

**Table 2:** Correlation statistics of miR-27a with disease symptoms and markers.

	miR-27a vs. DLCO	miR-27a vs. FVC	miR-27a vs. mRSS	miR-27a vs. ANA	miR-27a vs. Anti- dsDNA	miR-27a vs. TPO	miR-27a vs. anticcp	miR-27a vs. rf	miR-27a vs. crp
**Pearson r**									
r	0.000933	0.06084	− 0.08529	0.04795	− 0.03619	0.9202	− 0.01608	0.134	− 0.1279
95% confidence interval	− 0.2669 to 0.2686	− 0.2103 to 0.3233	− 0.3476 to 0.1894	− 0.2851 to 0.3706	− 0.5080 to 0.4523	0.6908 to 0.9813	− 0.2826 to 0.2528	− 0.1387 to 0.3879	− 0.3826 to 0.1448
R squared	8.71E−07	0.003702	0.007274	0.002299	0.00131	0.8467	0.000259	0.01796	0.01636
**P value**									
P (two-tailed)	0.9947	0.6621	0.5437	0.7813	0.8903	0.0002	0.9081	0.3339	0.3566
P value summary	ns	ns	ns	ns	ns	***	ns	ns	ns
Significant? (alpha = 0.05)	No	No	No	No	No	Yes	No	No	No

**Figure 4:**
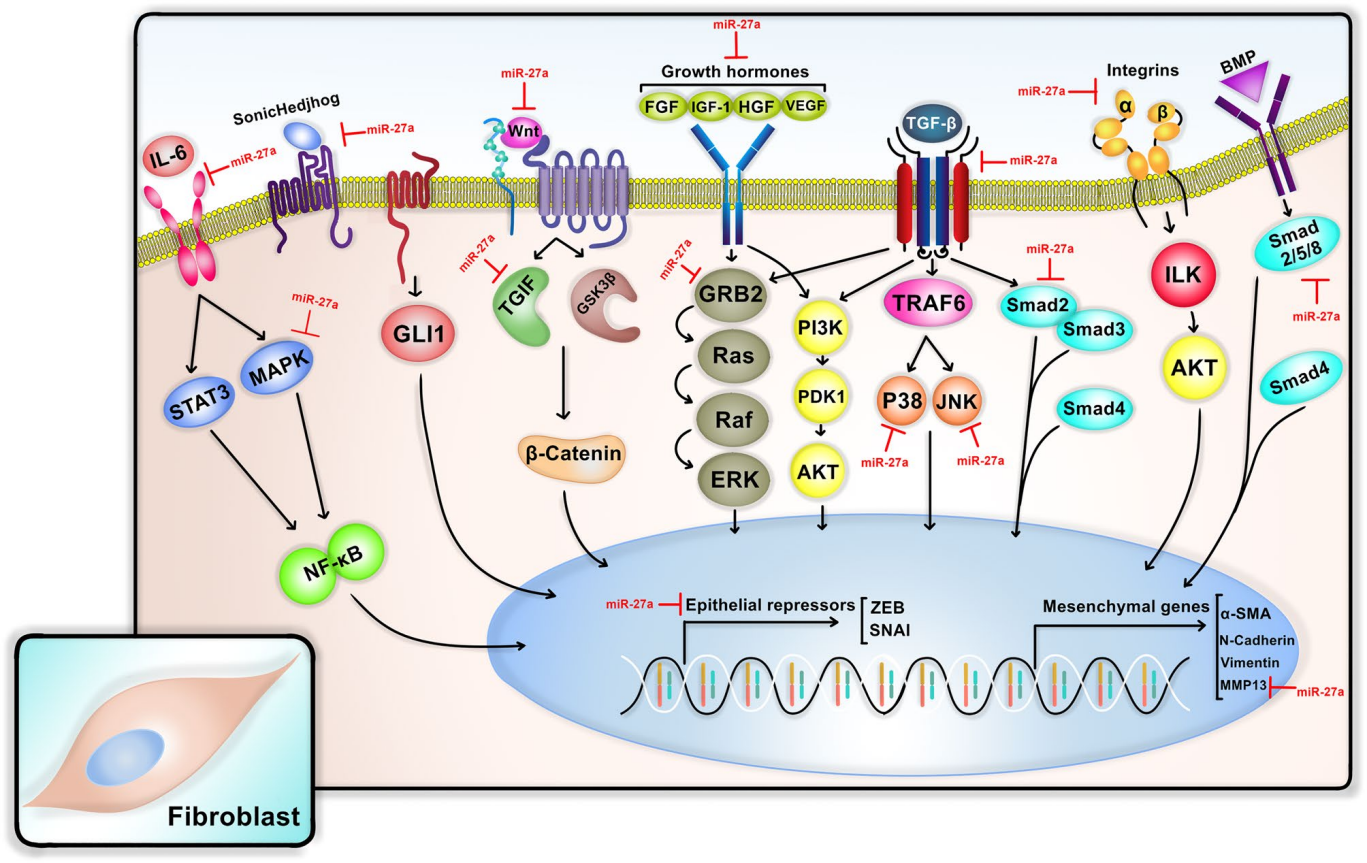
Predicted targets of miR-27a in the EMT pathway. miR-27a could interact with and target multiple gene transcripts contributing to the EMT signaling pathway which eventually results in the differentiation of fibroblasts into myofibroblasts; these cells are responsible for the development of fibrosis which is a key feature of SSc.

TargetScan (http://www.targetscan.org) predicted 1613 genes to be possibly targeted by miR-27a, among those genes, we searched for the genes involved in the several signaling pathways working downstream of the TGF-β which nearly regulates all aspects of fibrogenesis during a healing response, including but not limited to endothelial or epithelial to mesenchymal transition. Especially, the PI3K/AKT/mTOR pathway was considered for bioinformatics evaluations, as this pathway modulates many aspects of cell biology, such as proliferation, survival and protein synthesis; all of which are known to be involved in the pathogenesis of cancer and fibrosis. The genes were identified based on previous studies and data from KEGG as well as miRPathDB. It was found that miR-27a could target and regulate the expression of over 67 genes, known to be cardinals of EMT responsible for the transdifferentiation of fibroblasts into myofibroblasts; these cells overproduce high amounts of extracellular matrix components, including collagen and matrix metalloproteinases (MMPs). Table 3 lists the 67 genes predicted to be targeted by miR-27a, and Fig. 4 schematically illustrates these genes and their role in the development of fibrosis in SSc.

**Table 3:** The genes involved in EMT/fibrosis pathway, which were predicted to be targeted and regulated by miR-27a. The genes are sorted based on their targeting score.

Number	Gene symbol	Gene description
1	ROR1	Receptor tyrosine kinase-like orphan receptor 1
2	PPARG	Peroxisome proliferator-activated receptor gamma
3	FLRT3	Fibronectin leucine rich transmembrane protein 3
4	ACTA2	Actin, alpha 2, smooth muscle, aorta
5	VEGFC	Vascular endothelial growth factor C
6	SMAD9	SMAD family member 9
7	PDK4	Pyruvate dehydrogenase kinase, isozyme 4
8	FRS3	Fibroblast growth factor receptor substrate 3
9	MMP16	Matrix metallopeptidase 16
10	BMPR2	Bone morphogenetic protein receptor, type II
11	DKK2	Dickkopf WNT signaling pathway inhibitor 2
12	PDPK1	3-Phosphoinositide dependent protein kinase-1
13	TAB3	TGF-beta activated kinase 1/MAP3K7 binding protein 3
14	COL21A1	Collagen, type XXI, alpha 1
15	MAP3K4	Mitogen-activated protein kinase kinase kinase 4
16	VEGFB	Vascular endothelial growth factor B
17	SMAD5	SMAD family member 5
18	ITGA8	Integrin, alpha 8
19	SMURF2	SMAD specific E3 ubiquitin protein ligase 2
20	PDK1	Pyruvate dehydrogenase kinase, isozyme 1
21	PDGFRA	Platelet-derived growth factor receptor, alpha polypeptide
22	FGF14	Fibroblast growth factor 14
23	FOXO1	Forkhead box O1
24	FNDC4	Fibronectin type III domain containing 4
25	IRS1	Insulin receptor substrate 1
26	ITGA2	Integrin, alpha 2 (CD49B, alpha 2 subunit of VLA-2 receptor)
27	ELFN2	Extracellular leucine-rich repeat and fibronectin type III domain containing 2
28	WNT3A	Wingless-type MMTV integration site family, member 3A
29	SNAI1	Snail family zinc finger 1
30	FLRT2	Fibronectin leucine rich transmembrane protein 2
31	WNT2B	Wingless-type MMTV integration site family, member 2B
32	GAREM	GRB2 associated regulator of MAPK1
33	TGFBR1	Transforming growth factor, beta receptor 1
34	FZD3	Frizzled family receptor 3
35	FGF1	Fibroblast growth factor 1
36	MAPK10	Mitogen-activated protein kinase 10 (JNK)
37	TGFBR3	Transforming growth factor, beta receptor III
38	IL10	Interleukin 10
39	GSK3B	Glycogen synthase kinase 3 beta
40	HGF	Hepatocyte growth factor
41	MAP2K7	Mitogen-activated protein kinase kinase 7
42	MAPK8IP3	Mitogen-activated protein kinase 8 interacting protein 3
43	MMP13	Matrix metallopeptidase 13
44	TRAF3	TNF receptor-associated factor 3
45	ITGB8	Integrin, beta 8
46	FN1	Fibronectin 1
47	PPARGC1B	Peroxisome proliferator-activated receptor gamma, coactivator 1 beta
48	MAPK14	Mitogen-activated protein kinase 14 (p38)
49	HYOU1	hypoxia up-regulated 1
50	ITGA1	Integrin, alpha 1
51	FZD4	Frizzled family receptor 4
52	IL6ST	Interleukin 6 signal transducer (gp130, oncostatin M receptor)
53	FNDC3A	Fibronectin type III domain containing 3A
54	SIX1	SIX homeobox 1
55	COL5A1	Collagen, type V, alpha 1
56	MAPKAPK3	Mitogen-activated protein kinase-activated protein kinase 3
57	BMPR1A	Bone morphogenetic protein receptor, type IA
58	TAB2	TGF-beta activated kinase 1/MAP3K7 binding protein 2
59	SHH	Sonic hedgehog
60	BMP3	Bone morphogenetic protein 3
61	IGF1	Insulin-like growth factor 1 (somatomedin C)
62	FOXO3	Forkhead box O3
63	TGIF2	TGFB-induced factor homeobox 2
64	ITGA5	Integrin, alpha 5 (fibronectin receptor, alpha polypeptide)
65	COL11A2	Collagen, type XI, alpha 2
66	SMAD2	SMAD family member 2
67	TGFBR2	Transforming growth factor, beta receptor II

## Discussion

Fibrosis-associated disorders like systemic sclerosis (SSc) annually cause the morbidity and mortality of thousands of people around the world, yet there is a lot to be understood to unravel the exact mechanisms involved and hence no curative treatment plan exists for diseases like SSc. The broad range of symptoms and complications and also disease severity are among the main causes that this illness is not fully uncovered. Of the new research targets are microRNAs, which are implicated in all aspects of cell biology, and their roles in different diseases are of significant interest to scientists. Based on the previous studies regarding the role of miR-27 in cancer and fibrosis, which share many similar pathologic aspects, and since this microRNA is implicated in the regulation of the TGF-β signaling pathway as a cardinal regulator of fibrosis, we sought to assess the expression of miR-27 in whole blood of the SSc patients to evaluate its usefulness in the early diagnosis of SSc patients. Also, we aimed to evaluate its capability for distinguishing limited and diffused subsets of the disease, if applicable. The role of miRNAs in fibrosis-associated disorders has been widely studied, and it has been shown that miRNAs can target different aspects of fibrotic procedures^6,33,34^.

In the current study, the expression of miR-27 in the whole blood of SSc patients was investigated by taking advantage of Real-time qPCR; the statistical analyses demonstrated a significant downregulation of miR-27 in the SSc patients compared with the healthy controls. The expression of miR-27a was also slightly lower in the diffuse group compared with the limited group. According to the ROC analysis, a highly specific and sensitive cutoff was assigned to the qPCR data, suggesting an excellent diagnostic value of miR-27a for diagnosing SSc. We had presumed that the relative expression of miR-27 in diffuse SSc patients would be significantly different from the limited ones, but the results were not different significantly. In further analyses, the expression of miR-27a was compared between different subsets of patients based on the presence or absence of a specific condition or a laboratory marker. It was observed that the expression of miR-27a among those patients who were positive for anti-topoisomerase (anti-SCL70) antibody was significantly downregulated compared to the patients negative for this marker. As this marker significantly correlates with disease severity (diffuse form) and pulmonary involvement, this finding could suggest the potential of miR-27a for differentiating patients from healthy ones as well as utilizing it in the assessment of disease progression, although this hypothesis needs further robust examinations in much larger populations of the SSc patients to draw a comprehensive conclusion. Also, the expression of miR-27a was evaluated in subsets of patients positive for ACA and ribonucleoprotein autoantibodies; it was found that the downregulation of miR-27a is associated with negative ACA, which can be explained by the very low frequency of ACA in Iranian SSc patients, also ACA is more prevalent among the limited subtype of the SSc and this is consistent with our data^35–37^. Besides, it was shown that the downregulation of miR-27a which is found in diffuse patients is also associated with positive anti-ribonucleoprotein antibodies. Disease manifestations such as ILD, PAH, myositis, and digital ulcers were all associated with the downregulation of miR-27a which is in line with previous results, as the downregulation of miR-27a is associated with the diffuse subtype and the complications are more prevalent among this subset. On the other hand, the downregulation of miR-27a was associated with the absence of telangiectasia. Also, it seems that the expression of miR-27a in male patients is higher than the female one. One major limitation of our data is that there were no recently diagnosed patients in our study, and the majority had an average five years history of being diagnosed with SSc. Due to the challenges regarding the diagnosis of these patients in the early stages, we were unable to find a minimum number of newly diagnosed patients required for obtaining statistically significant results.

As illustrated in Fig. 4, miR-27a was predicted to play an essential role in the fibrosis process. This microRNA targets various gene transcripts downstream of the TGF-β pathway which are involved in the transdifferentiation of fibroblasts into myofibroblasts; this process serves as the basis for the development of fibrosis-associated complications, including skin thickening, vascular problems, and organ fibrosis. It should be noted that the epithelial-to-mesenchymal transition (EMT) process in cancer refers to the transition of epithelial cells into ECM-secreting mesenchymal cells but in the course of fibrotic procedures, fibroblasts transformation into myofibroblasts is called fibroblast-to-myofibroblast transition or transdifferentiation. In this regard, we found different studies, which confirm the inhibitory effect of miR-27a on the predicted genes. It was shown that miR-27a suppresses inflammation by targeting IL-6 and MAP kinases like P38 and JNK^38^. Besides, miR-27a was found to inhibit the expression of SMAD signaling mediators that act downstream of the TGF-β pathway^39^. Another inhibitory effect of miR-27a on the TGF-β signaling pathway is mediated via direct targeting of the TGF-βRI transcript^22^. Also, major growth factors capable of contributing to fibrosis pathogenesis were found to be downregulated by miR-27a, including IGF-1 and VEGF^40,41^. All of the aforementioned studies are in line with our results which strongly demonstrate the protective effect of miR-27a against fibrosis and hence SSc. As depicted in Fig. 4, miR-27a also targets and regulates MMP13 which is a matrix metalloproteinase involved in the remodeling of extracellular matrix during fibrotic changes^42^. Recently, Qi Cheng et al. have shown that miR-27a is capable of suppressing fibrosis in vitro by targeting the secreted phosphoprotein 1 (SPP1) as well^43^.

MiR-27a has been studied widely in cancer and metabolism^44^, but to our knowledge, this is the first study considering the role of miR-27a in systemic sclerosis, although it needs further functional analyses in order to give a comprehensive and conclusive outcome. The remarkable finding of our study is the downregulation of miR-27a in the whole blood of patients affected with systemic sclerosis (either limited or diffused) in comparison to healthy individuals. Thus, we could propose a role for miR-27a in SSc; it seems likely that miR-27 negatively affects molecular pathways involved in fibrogenesis, such as the TGF-β signaling pathway, and as a result, it could be considered an anti-fibrotic microRNA. In support of our theory Cui H, et al. investigated the therapeutic effect of miR-27a on pulmonary fibrosis by demonstrating the inhibitory effect of miR-27a on Smad2 and Smad4, which are the major mediators of the TGF-β signaling pathway; They have shown that α-smooth muscle actin is directly regulated by miR-27a as well, which is the characteristic feature of myofibroblast differentiation^28^. Also, there are other shreds of evidence regarding the relationship between miR-27a and the TGF-β signaling pathway in other circumstances. Consistent with our theory, Fang Fang, et al. have shown the ability of miR-27a to inhibit the TGF-β signaling pathway through suppression of TGF-βRI, Smad2, and Smad4 expression; as well as reduced phosphorylation of Smad3 in cervical adenocarcinoma; cell proliferation, migration, and invasion which employ the same mechanism of EMT in fibrosis, were also attenuated^22^. Dong-Kyu Chae et al. have shown the negative regulation of SMAD2, and SMAD4 by miR-27a in lung cancer cell lines^45^. Consistently, Qi Xu, et al. have demonstrated that miR-27a suppresses TGF-β-induced expression of SMAD4 in human lymphatic endothelial cells, which supports our hypothesis on the role of miR-27a as a likely anti-fibrotic factor^46^. It is noteworthy to mention that in a model of myocardial ischemia, Zhang Xl, et al. have shown the downregulation of TGFβRI along with the reduction of IL-6, TNF-α, and p-NFκB by miR-27a^47^; all of which are major inflammatory factors contributing to fibrosis pathogenesis. On the contrary, Hui Zhang, et al. have investigated the role of miR-27a in hepatic stellate cell (HSC ) activation and fibrosis and concluded that miR-27a increases in response to TGF-β and contributes to HSC activation and fibrosis; they have demonstrated that the inhibition of miR-27a leads to the suppression of PPARγ, α-SMA and collagen too^48^.Quin Lin, Et al. and Sun Young Jang, et al. in two different but similar studies on adipogenesis, have shown that the overexpression of miR-27a results in the downregulation of PPARγ, which is known as an inhibitor of TGF-β non-canonical pathway^25,49^. In this regard, Deng K, et al. have shown the negative regulation of PPARγ by miR-27a, too^50^. Besides, in a study conducted by Ji-Hoon Cho, et al. with a systems biology approach, it was found that miR-27a along with miR-23a and miR24 is upregulated in response to TGF-β by the Zeb-1 transcription factor which is a major participant in EMT^35^. It could be suggested that miR-27a elevation in these instances may take place upon over-activation of the TGF-β signaling pathway as a negative feedback mechanism to attenuate its physiological consequences. The other regulator of the TGF-β pathway, PTEN, has also been considered as a target of miR-27a; In an interesting study, though on diabetic mice models, Aiqing Zhang, et al. have shown the ability of miR-23a-mir-27a cluster for downregulation of FoxO1 and PTEN; they have also demonstrated that the expression of TGF-β and pSMAD3/4 are suppressed by miR-27a thereby resulting in the attenuation of renal fibrosis^51^. Collectively we believe that miR-27a has a regulatory role regarding the signaling pathways involved in fibrosis and therefore SSc, and the majority of data support the idea that miR-27a has a protective effect against SSc. Also, it is likely that a genetic deficit or any contributing factor for SSc causes the downregulation of miR-27a thereby leading to the aberrant activation of TGF-β and other signaling pathways contributing to SSc pathogenesis. The major limitation of our study is lack of functional assays such as utilizing miR-27a mimics or anatgomiRs in cultures of cells obtained from the SSc patients and assessing the alterations of the possible targets to further confirm the results of the current srudy, which we hope to be addressed in near future.

## Conclusion

Based on the statistical analyses, miR-27a could serve as a reliable diagnostic marker for SSc, and given its proposed role in regulating TGF-β and other contributing pathways to SSc, it could be considered as a treatment option both for SSc and its related disorders and complications, which indeed necessitates further investigations.

## Data Availability

The datasets used and/or analyzed during the current study are available from the corresponding author upon reasonable request.
